# Variances and covariances in the Central Limit Theorem for the output of a transducer

**DOI:** 10.1016/j.ejc.2015.03.004

**Published:** 2015-10

**Authors:** Clemens Heuberger, Sara Kropf, Stephan Wagner

**Affiliations:** a Institut für Mathematik, Alpen-Adria-Universität Klagenfurt, Austria; b Department of Mathematical Sciences, Stellenbosch University, South Africa

## Abstract

We study the joint distribution of the input sum and the output sum of a deterministic transducer. Here, the input of this finite-state machine is a uniformly distributed random sequence.

We give a simple combinatorial characterization of transducers for which the output sum has bounded variance, and we also provide algebraic and combinatorial characterizations of transducers for which the covariance of input and output sum is bounded, so that the two are asymptotically independent.

Our results are illustrated by several examples, such as transducers that count specific blocks in the binary expansion, the transducer that computes the Gray code, or the transducer that computes the Hamming weight of the width-w non-adjacent form digit expansion. The latter two turn out to be examples of asymptotic independence.

## 1 Introduction

We consider sequences defined as the sum of the output of a deterministic transducer, i.e. a finite-state machine that deterministically transforms an input sequence into an output sequence. Here, we let both the input and the output be sequences of real numbers and assume that the input sequence is randomly generated. Then, while the output depends deterministically on the input, the dependence between the two random variables “sum of the input sequence” and “sum of the output sequence” may become negligible for long input sequences. We investigate for which transducers this is the case. We give two different characterizations of such “independent” transducers, an algebraic and a combinatorial one. In a similar way, we also consider the variance of the sum of the output of a transducer. We prove a combinatorial characterization of transducers with bounded variance of the output sum. These combinatorial characterizations are described in terms of a weighted number of functional digraphs or cycles of the underlying graph.

Our probability model is the equidistribution on all input sequences of a fixed length n. We asymptotically investigate the two random variables “sum of the input” and “sum of the output” of a transducer for n→∞. If these two random variables converge in distribution to independent random variables, then the transducer is called independent.

Under this probability model, the expected value of the sum of the input and the output are $e_{1}n$ and $e_{2}n+O\left(1\right)$, respectively, for some constants $e_{1}$ and $e_{2}$. For the sum of the input, the expressions are exact without error term because the input letters are independent and identically distributed. Furthermore, under appropriate connectivity conditions, the variances and the covariance turn out to be $v_{1}n$, $v_{2}n+O\left(1\right)$ and cn+O(1), respectively, for suitable constants $v_{1}$, $v_{2}$ and c. We investigate for which transducers one of the constants $v_{2}$ and c is zero.

A special case of the output sum is the Hamming weight, which is the number of nonzero elements of a sequence. To give an example of an independent transducer, we discuss the Hamming weight of the non-adjacent form as defined by Reitwiesner  [24] in Example 2.5. In  [19], Heuberger and Prodinger prove that the Hamming weights of the standard binary expansion and the non-adjacent form are asymptotically independent. The independent transducer computing these Hamming weights is shown in Fig. 2.

There are many results on the variance of the sum of the output of explicit transducers under the same probability model we use. See, for example,  [9], [10], [2], [13] for the variance of the Hamming weight of different digit expansions which are computed by transducers. In  [18], the authors count the occurrences of a digit and give the expected value, the variance and the covariance between two different digits. The occurrence of a specific pattern in a word is investigated in e.g.  [3], [23], [6], [8] (with generalizations to other probability models, too). In  [3], the covariance between different patterns is also considered. In  [11], Grabner and Thuswaldner consider a transducer whose output is the sum of digit function. However, they were only interested in the output and did not consider the joint distribution or the covariance of the input and output sum.

By contrast, we are interested in the joint distribution of the input and output sum for a general transducer. We not only algebraically compute the expected value and the variance–covariance matrix of this distribution, but we also give combinatorial descriptions of these values. In particular, we combinatorially characterize independent transducers and transducers with bounded variance of the output sum. This combinatorial connection is described by a condition on some weighted number of functional digraphs or on each cycle of the underlying graph of the transducer. To obtain these results, we apply a generalization of the Matrix-Tree Theorem by Chaiken  [4] and Moon  [21].

We formally define our setting in the next section. In Section  3, we state our main results. In Section  4, we present several examples where these main results are applied. In the last section, we give the proofs of the theorems.

In many contexts, an unbounded variance (as in  [20]) is necessary to prove a Gaussian limit law. In Theorem 3.1, we combinatorially describe transducers whose output sums have bounded variance. For strongly connected transducers, we prove that this is the case if and only if there exists a constant such that for each cycle, the output sum is proportional to its length with this proportionality constant. This in turn is equivalent to a quasi-deterministic output sum in the sense that the difference of the output sum and its expected value is bounded for *all* events, independently of the length of the input. In the special case where the transducer is strongly connected and aperiodic and the only possible outputs are 0 and 1, it turns out that the output sum has asymptotically bounded variance if and only if the output is constant for all transitions (Corollary 3.6). The assumption of strong connectivity can be relaxed for most results.

We give an algebraic description of independent transducers in Theorem 3.9. We also state there that the input sum and the output sum are asymptotically jointly normally distributed if the variance–covariance matrix is invertible. In Theorem 3.14, we present a combinatorial characterization of independent transducers.

In Section  4, we give a variety of examples of independent and dependent transducers and transducers with bounded and unbounded variance to illustrate our results. One of those examples is a transducer computing the minimal Hamming weight of τ-adic digit representations on a digit set D. Building on the results of  [13], we prove that the variance of the minimal Hamming weight is unbounded, which yields a central limit theorem.

In Section  5, we also prove an extension of the 2-dimensional Quasi-Power Theorem  [14] to singular Hessian matrices as an auxiliary result.

The results of Theorem 3.9 have been implemented  [17] in the open-source mathematics software system SageMath   [26], based on its package for finite state machines  [15]. This code is included in SageMath  6.3.

## 2 Preliminaries

A *transducer* is defined to consist of a finite set of states {1,2,…,S}, a finite input alphabet $A_{I}\subseteq R$, an output alphabet $A_{O}\subseteq R$, a set of transitions $E\subseteq {\left\{1,2,\ldots ,S\right\}}^{2}\times A_{I}$ with input labels in $A_{I}$, output labels $\delta :E\to A_{O}$ and the initial state 1. The transducer is called *deterministic* if for all states s and input labels $\varepsilon \in A_{I}$, there exists at most one state t such that (s,t,ε)∈E. Furthermore, the transducer is said to be *subsequential* (cf.  [25]) if it is deterministic, every state is final and it has a final output $a:\left\{1,2,\ldots ,S\right\}\to A_{O}$. A transducer is called *complete* if for every state s and digit $\varepsilon \in A_{I}$, there is a transition from s to a state t with input label ε, i.e., (s,t,ε)∈E.

Definition 2.1 A transducer is said to be *finally connected* if there exists a state which can be reached from any other state. The *final component* of such a transducer is defined to be the transducer induced by the set of states which can be reached from any other state. A finally connected transducer is said to be *finally aperiodic* if the underlying graph of the final component is aperiodic (i.e., the gcd of the lengths of all walks starting and ending at a given vertex is 1).

Remark 2.2 The final component of a transducer is a strongly connected component of the underlying graph of the transducer. If the underlying graph is strongly connected, then being finally aperiodic is equivalent to being aperiodic. We then call the transducer strongly connected and aperiodic. The final component of a complete transducer is complete itself.

In the following, we consider subsequential, complete, deterministic, finally connected, finally aperiodic transducers. We require that the input alphabet $A_{I}$ has at least two elements. Throughout the paper, we use ε for the input of a transition and δ for the output of a transition. We denote the number of states in the final component by N.

The input of the transducer is a sequence in $A_{I}^{*}$. It is not important whether we read the input from right to left or in the other direction, we just have to fix it for one specific transducer. The output of the transducer is the sequence of output labels of the unique path starting at the initial state 1 with the given input as input label, together with the final output label of the final state of this path.

Let $X_{n}$ be a uniformly distributed random variable on $A_{I}^{n}$. Let $\mathrm{Output}\left(X_{n}\right)$ be the sum of the output sequence of the transducer if the input is $X_{n}$. Furthermore, let $\mathrm{Input}\left(X_{n}\right)$ be the sum of the input sequence. Without loss of generality, we fix the direction of reading from right to left.

**Fig. 1:**
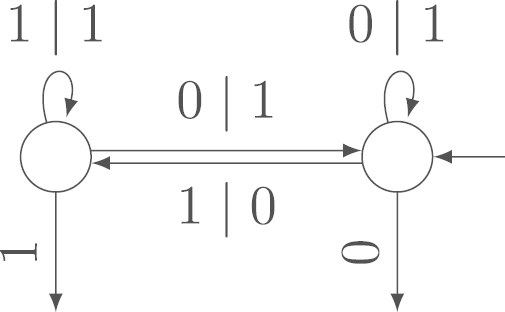
The subsequential, complete, strongly connected, aperiodic transducer from Example 2.3.

Example 2.3 The transducer in Fig. 1 is a subsequential, complete, strongly connected, aperiodic transducer.

For example, when reading the input (110) from right to left, the transducer in Fig. 1 writes the output (1101). The leftmost 1 in the output is the final output of the last state. The output sum is Output(110)=3.

We investigate the 2-dimensional random vector

$$\Omega_{n}={\left(\mathrm{Input}\left(X_{n}\right),\mathrm{Output}\left(X_{n}\right)\right)}^{t}$$

for n→∞, where ${}^{t}$ denotes transposition. We will prove that each component of this random vector either converges in distribution to a normally distributed random variable or to a degenerate random variable. Here, a random variable is said to be *degenerate* if it is constant with probability 1. By definition, a degenerate random variable is independent of any other random variable. Thus, the variance of a degenerate random variable and the covariance of a degenerate and any other random variable are always 0.

For a finally connected, aperiodic transducer, the expected value and the variance of $\Omega_{n}$ will turn out to be ${\left(e_{1},e_{2}\right)}^{t}n+O\left(1\right)$, ${\left(v_{1},v_{2}\right)}^{t}n+O\left(1\right)$, respectively, for suitable constants $e_{1}$, $e_{2}$, $v_{1}$ and $v_{2}$ (see Theorem 3.9). The covariance between the two coordinates will be cn+O(1) for some constant c. We call $\Sigma =\left(\begin{array}{cc} v_{1} & c\\ c & v_{2} \end{array}\right)$ the *asymptotic variance–covariance matrix* of $\Omega_{n}={\left(\mathrm{Input}\left(X_{n}\right),\mathrm{Output}\left(X_{n}\right)\right)}^{t}$. Its entries are called the *asymptotic variances* and the *asymptotic covariance*.

For transducers with output alphabet {0,1}, the characterization of vanishing asymptotic variance $v_{2}$ turns out to be particularly simple: All transitions of the final component have to have the same output (see Corollary 3.6). This output alphabet occurs naturally when only the Hamming weight (the number of nonzero elements) of an expansion is of interest.

For brevity, we introduce the notion of independent transducers.

Definition 2.4 A transducer is *independent* if the random vector $\Omega_{n}$ converges in distribution to a random vector with two independent components, i.e., the sum of the input $\mathrm{Input}\left(X_{n}\right)$ and the sum of the output $\mathrm{Output}\left(X_{n}\right)$ are asymptotically independent random variables.

Example 2.5 In  [19], Heuberger and Prodinger prove that the Hamming weight of the standard binary expansion and the Hamming weight of the non-adjacent form are asymptotically independent. The non-adjacent form is the unique digit expansion with digits {−1,0,1}, base 2 and the syntactical rule that at least one of any two adjacent digits has to be 0. It has minimal Hamming weight among all digit expansions with digits {−1,0,1} in base 2.

**Fig. 2:**
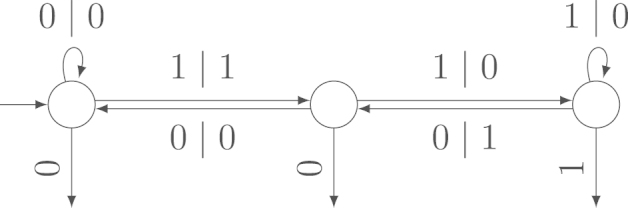
Transducer to compute the Hamming weight of the non-adjacent form.

The transducer in Fig. 2 computes the Hamming weight of the non-adjacent form when reading the binary expansion from right to left. The transducer is a slight simplification of the one in, e.g.,  [19], taking into account that we are only interested in the Hamming weight. Thus, the transducer in Fig. 2 is an example of an independent transducer by the results in  [19].

## 3 Main results

In this section, we state the main theorems and corollaries describing independent transducers and transducers with bounded variance. First, we investigate transducers with bounded variance. Then, we give an algebraic description and a combinatorial characterization of independent transducers. All proofs can be found in Section  5.

### 3.1 Bounded variance and singular asymptotic variance–covariance matrix

We give a combinatorial characterization of transducers whose output sum has asymptotic variance 0. We also give a combinatorial description of transducers with singular asymptotic variance–covariance matrix. These characterizations are given in terms of cycles and closed walks of directed graphs.

As usual, a *cycle* is a strongly connected digraph such that every vertex has out-degree 1. A *closed walk* is an alternating sequence of vertices and edges $\left(s_{1},e_{1},s_{2},\ldots ,s_{n+1}=s_{1}\right)$ such that $e_{j}$ is an edge from $s_{j}$ to $s_{j+1}$.

For a function g and a walk C of the underlying graph of the transducer, we define

$$g\left(C\right)=\underset{e\in C}{\sum }g\left(e\right)$$

taking multiplicities into account. Here, the function g is either the constant function 1(e)=1, the input ε(e) or the output δ(e) of the transition e.

Theorem 3.1 *For a subsequential, complete, finally connected and finally aperiodic transducer with an arbitrary finite input alphabet* $A_{I}$*, the following assertions are equivalent:*

(a) *The asymptotic variance* $v_{2}$ *of the output sum is*  0*.*
(b) *There exists a state* s *of the final component and a constant* k∈R *such that*
  δ(C)=k1(C)
  *holds for every closed walk* C *of the final component visiting the state* s *exactly once.*
(c) *There exists a constant* k∈R *such that*
  δ(C)=k1(C)
  *holds for every directed cycle* C *of the final component of the transducer* T*.*

*In that case,* kn+O(1) *is the expected value of the output sum and Statement*  (b)  *holds for all states* s *of the final component.*

We want to emphasize that only cycles and closed walks of the final component are considered in this theorem (see also Remark 3.10). The proofs can be found in Section  5.3.

In the case of a strongly connected transducer, the equivalent conditions of Theorem 3.1 will be shown to be equivalent to another condition which, at first glance, seems to be even stronger.

Definition 3.2 The output sum of a transducer is called *quasi-deterministic* if there is a constant k∈R such that

$$\mathrm{Output}\left(X_{n}\right)=kn+O\left(1\right)$$

holds for all n and all inputs.

We now characterize quasi-deterministic output sums. In weakly connected graphs, it turns out that being “quasi-deterministic” is a stronger notion than the conditions in Theorem 3.1.

Theorem 3.3 *Let* T *be a subsequential, complete transducer whose underlying graph is weakly connected. Then the following two assertions are equivalent:*

(d) *There exists a constant* k∈R *such that the random variable* $\mathrm{Output}\left(X_{n}\right)$ *is quasi-deterministic with value* kn+O(1)*.*
(e) *There exists a constant* k∈R *such that*
  δ(C)=k1(C)
  *holds for every directed cycle* C *of the transducer.*

This result and the following corollaries are proved in Section  5.3. By comparing statements (c) of Theorem 3.1 and (e) of Theorem 3.3, it is obvious that in strongly connected transducers, all these statements are actually equivalent.

Corollary 3.4 *Let* T *be a subsequential, complete, strongly connected, aperiodic transducer. Then the asymptotic variance* $v_{2}$ *of the output sum is zero if and only if the output sum is a quasi-deterministic random variable.*

Remark 3.5 If the transducer is not strongly connected (so that there are states that do not belong to the final component), the output sum can have bounded variance without being quasi-deterministic. A simple example is a transducer that counts the number of 1s in a binary string before the first 0. In such a case, however, the transducer formed only by the final component still needs to have quasi-deterministic output sum.

When considering the special case of the Hamming weight, bounded variance only occurs in trivial cases:

Corollary 3.6 *For* $A_{O}=\left\{0,1\right\}$*, the only output weights of the final component with asymptotic variance* $v_{2}=0$ *are* (0,…,0) *and* (1,…,1)*.*

The following corollary of Theorem 3.1 gives a combinatorial characterization of transducers whose asymptotic variance–covariance matrix is singular.

Corollary 3.7 *Let* T *be a complete, subsequential, finally connected, finally aperiodic transducer whose input alphabet has at least size*  2*. Then the asymptotic variance–covariance matrix* Σ *has rank*  1  *if and only if there exist* a*,* b∈R *with*

(1) δ(C)=a1(C)+bε(C)

*for all cycles* C *of the final component.*

*In that case, the constants are* $a=-\frac{c}{v_{1}}e_{1}+e_{2}$ *and* $b=\frac{c}{v_{1}}$*.*

*Furthermore, the random variables* $\mathrm{Input}\left(X_{n}\right)$ *and* $\mathrm{Output}\left(X_{n}\right)$ *are asymptotically perfectly positively or negatively correlated (i.e., they have asymptotic correlation coefficient* ±1 *) if and only if*   (1)   *holds with* b≠0*.*

### 3.2 Algebraic description of independent transducers

For giving an algebraic description of independent transducers, we define transition matrices of the transducer.

Definition 3.8 For $\varepsilon \in A_{I}$, let a *transition matrix* $M_{\varepsilon }\left(y\right)$ of the final component be the N×N-matrix whose entry (s,t) is $y^{\delta }$ if there is a transition from state s to state t in the final component with input ε and output δ, and 0 otherwise.

Similarly, let $W_{\varepsilon }$ be the transition matrix of the whole transducer. The ordering of the states is considered to be fixed in such a way that the initial state 1 is the first state and $W_{\varepsilon }$ has the block structure

(2) $$\left(\begin{array}{cc} {}* & *\\ 0 & M_{\varepsilon } \end{array}\right)$$

where ∗ are matrices with arbitrary entries. If the transducer is strongly connected, the matrices ∗ are not present (they have 0 rows).

Theorem 3.9 *Let* T *be a complete, subsequential, finally connected, finally aperiodic transducer, and let the transition matrices of the final component be* $M_{\varepsilon }\left(y\right)$ *for* $\varepsilon \in A_{I}$ *. Set*

$$f\left(x,y,z\right)=\det\left(I-\frac{z}{\left|A_{I}\right|}\underset{\varepsilon \in A_{I}}{\sum }x^{\varepsilon }M_{\varepsilon }\left(y\right)\right)\text{.}$$

*Then the random variables* $\mathrm{Input}\left(X_{n}\right)$ *and* $\mathrm{Output}\left(X_{n}\right)$ *have the expected values, variances and covariance*

(3) $$E\left(\mathrm{Input}\left(X_{n}\right)\right)=e_{1}n\text{,}$$

$$E\left(\mathrm{Output}\left(X_{n}\right)\right)=e_{2}n+O\left(1\right)\text{,}$$

$$V\left(\mathrm{Input}\left(X_{n}\right)\right)=v_{1}n\text{,}$$

$$V\left(\mathrm{Output}\left(X_{n}\right)\right)=v_{2}n+O\left(1\right)\text{,}$$

$$\mathrm{Cov}\left(\mathrm{Input}\left(X_{n}\right),\mathrm{Output}\left(X_{n}\right)\right)=cn+O\left(1\right)$$

*with*

$$e_{1}=\frac{f_{x}}{f_{z}}{|}_{1}\text{,}$$

$$e_{2}=\frac{f_{y}}{f_{z}}{|}_{1}\text{,}$$

$$v_{1}=\frac{1}{f_{z}^{3}}\left(f_{x}^{2}\left(f_{zz}+f_{z}\right)+f_{z}^{2}\left(f_{xx}+f_{x}\right)-2f_{x}f_{z}f_{xz}\right){|}_{1}\text{,}$$

$$v_{2}=\frac{1}{f_{z}^{3}}\left(f_{y}^{2}\left(f_{zz}+f_{z}\right)+f_{z}^{2}\left(f_{yy}+f_{y}\right)-2f_{y}f_{z}f_{yz}\right){|}_{1}\text{,}$$

$$c=\frac{1}{f_{z}^{3}}\left(f_{x}f_{y}\left(f_{zz}+f_{z}\right)+f_{z}^{2}f_{xy}-f_{y}f_{z}f_{xz}-f_{x}f_{z}f_{yz}\right){|}_{1}$$

*where* $1={\left(1,1,1\right)}^{t}$ *and* $f_{z}\left(1\right)\neq 0$*.*

*The constants* $e_{1}$ *and* $v_{1}$ *can also be expressed as*

(4) $$e_{1}=\frac{1}{\left|A_{I}\right|}\underset{\varepsilon \in A_{I}}{\sum }\varepsilon \text{,}\;v_{1}=\frac{1}{\left|A_{I}\right|}\underset{\varepsilon \in A_{I}}{\sum }\varepsilon^{2}-{\left(\frac{1}{\left|A_{I}\right|}\underset{\varepsilon \in A_{I}}{\sum }\varepsilon \right)}^{2}\text{.}$$

*The random vector* $\Omega_{n}$ *is asymptotically jointly normally distributed if and only if the asymptotic variance–covariance matrix* Σ *is regular.*

*The transducer* T *is independent if and only if*

(5) $$\left(f_{x}f_{y}\left(f_{zz}+f_{z}\right)+f_{z}^{2}f_{xy}-f_{y}f_{z}f_{xz}-f_{x}f_{z}f_{yz}\right){|}_{1}=0$$

*or, equivalently,*

(6) $$\left(e_{1}f_{y}\left(f_{zz}+f_{z}\right)+f_{z}f_{xy}-f_{y}f_{xz}-e_{1}f_{z}f_{yz}\right){|}_{1}=0\text{.}$$

The proof of this theorem is in Section  5.1. This result has been implemented as the method

FiniteStateMachine.asymptotic_moments()

in the mathematics software system SageMath, cf.  [17], using the finite state machines package described in  [15].

Remark 3.10 Neither the final output nor the non-final components influence the asymptotic result because it only depends on f(x,y,z) and thus on the transitions of the final component.

Now we consider the following “inverse” problem: Given the underlying graph and the input digits of the transducer; how can we choose the output labels such that the transducer is independent?

Let $\left(a_{1},\ldots ,a_{N}\right)$ be the output labels of the final component of the transducer. We say, as usual, that a linear equation is homogeneous if the zero vector is a solution. Then  (5) is a linear, homogeneous equation in $a_{1},\ldots ,a_{N}$ with real coefficients. The equation is linear because the variables $a_{i}$ only occur linearly in the exponents of y and there are only first derivatives with respect to y in the covariance condition  (5). Furthermore,  (5) is homogeneous because all derivatives with respect to y (and maybe other additional variables) at ${\left(x,y,z\right)}^{t}=1$ are homogeneous. A solution of this linear, homogeneous equation corresponds to an independent transducer.

Let us first consider the situation where all outputs are equal to 1. Then, the determinant f(x,y,z) consists of monomials $x^{a}y^{b}z^{b}$ with a∈R and b∈Z. Therefore, we obtain

$$f_{y}{|}_{1}=f_{z}{|}_{1}\text{,}$$

$$f_{xy}{|}_{1}=f_{xz}{|}_{1}\text{,}$$

$$f_{yz}{|}_{1}=f_{zz}+f_{z}{|}_{1}\text{,}$$

and it follows that  (5), (6) are satisfied. This means that a constant output (k,…,k) for $k\in A_{O}$ is always a trivial solution to these equations because  (5) is homogeneous.

But for these trivial solutions, the sum of the output is an asymptotically degenerate random variable. Hence, we are not really interested in the independent transducers given by these solutions.

**Fig. 3:**
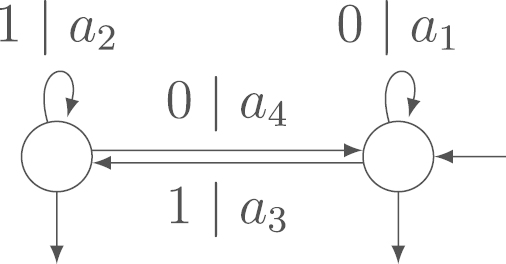
Transducer of Example 3.11.

Example 3.11 In Fig. 3, we have a transducer with variable output weights $a_{1}$, $a_{2}$, $a_{3}$ and $a_{4}$. We do not give the final output labels as they do not influence the asymptotic result. In this example,  (5) simplifies to

$$-a_{1}+a_{2}=0\text{.}$$

### 3.3 Combinatorial characterization of independent transducers

We connect the derivatives of f(x,y,z) with a weighted sum of subgraphs of the underlying graph. Thus, in Theorem 3.14, we can give a combinatorial description of  (5).

Definition 3.12 We define the following types of directed graphs as subgraphs of the final component of the transducer.

• A *rooted tree* is a weakly connected digraph with one vertex which has out-degree 0, while all other vertices have out-degree 1. The vertex with out-degree 0 is called the *root* of the tree.
• A *functional digraph* is a digraph whose vertices have out-degree 1. Each component of a functional digraph consists of a directed cycle and some trees rooted at vertices of the cycle. For a functional digraph D, let $C_{D}$ be the set of all cycles of D.

Definition 3.13 Let $D_{1}$ and $D_{2}$ be the sets of all spanning subgraphs of the final component of the transducer T which are functional digraphs and have one and two components, respectively.

For functions g and h:E→R, we define

$$g\left(D_{1}\right)=\underset{D\in D_{1}}{\sum }\underset{C\in C_{D}}{\sum }g\left(C\right)\text{,}$$

$$gh\left(D_{1}\right)=\underset{D\in D_{1}}{\sum }\underset{C\in C_{D}}{\sum }g\left(C\right)h\left(C\right)\text{,}$$

gh(D2)=∑D∈D2∑C1∈CD∑C2∈CDC2≠C1g(C1)h(C2).

With these definitions, we give a combinatorial characterization of independent transducers.

Theorem 3.14 *Let* T *be a complete, subsequential, finally connected, finally aperiodic transducer.*

*Then the random variables* $\mathrm{Input}\left(X_{n}\right)$ *and* $\mathrm{Output}\left(X_{n}\right)$ *have the expected values given by*   (3)*, where the constants are*

$$e_{1}=\frac{\varepsilon \left(D_{1}\right)}{1\left(D_{1}\right)}\text{,}$$

$$e_{2}=\frac{\delta \left(D_{1}\right)}{1\left(D_{1}\right)}\text{.}$$

*The variances and the covariance are given by*   (3)*, with the constants*

$$v_{1}=\frac{1}{1\left(D_{1}\right)}\left(\left(\varepsilon -e_{1}1\right)\left(\varepsilon -e_{1}1\right)\left(D_{1}\right)-\left(\varepsilon -e_{1}1\right)\left(\varepsilon -e_{1}1\right)\left(D_{2}\right)\right)\text{,}$$

$$v_{2}=\frac{1}{1\left(D_{1}\right)}\left(\left(\delta -e_{2}1\right)\left(\delta -e_{2}1\right)\left(D_{1}\right)-\left(\delta -e_{2}1\right)\left(\delta -e_{2}1\right)\left(D_{2}\right)\right)\text{,}$$

$$c=\frac{1}{1\left(D_{1}\right)}\left(\left(\varepsilon -e_{1}1\right)\left(\delta -e_{2}1\right)\left(D_{1}\right)-\left(\varepsilon -e_{1}1\right)\left(\delta -e_{2}1\right)\left(D_{2}\right)\right)\text{.}$$

*The transducer* T *is independent if and only if*

(7) $$\left(\varepsilon -e_{1}1\right)\left(\delta -e_{2}1\right)\left(D_{1}\right)=\left(\varepsilon -e_{1}1\right)\left(\delta -e_{2}1\right)\left(D_{2}\right)\text{.}$$

We emphasize that, by Definition 3.13, only edges in the final component of the transducer are considered in Theorem 3.14. The non-final components do not influence the asymptotic main terms (see also Remark 3.10). The proof of this theorem can be found in Section  5.2.

In the following corollary, we consider the case of a normalized input and output, i.e., the constants of the expected values satisfy $e_{1}=e_{2}=0$. This can be obtained by subtracting the original constants $e_{1}$ and $e_{2}$ from every input label and output label, respectively. Then the corollary follows directly from Theorem 3.14.

Corollary 3.15 *Suppose that* $E\left(\mathrm{Input}\left(X_{n}\right)\right)$ *and* $E\left(\mathrm{Output}\left(X_{n}\right)\right)$ *are both bounded. Then the transducer* T *is independent if and only if*

$$\varepsilon \delta \left(D_{2}\right)=\varepsilon \delta \left(D_{1}\right)\text{.}$$

**Fig. 4:**
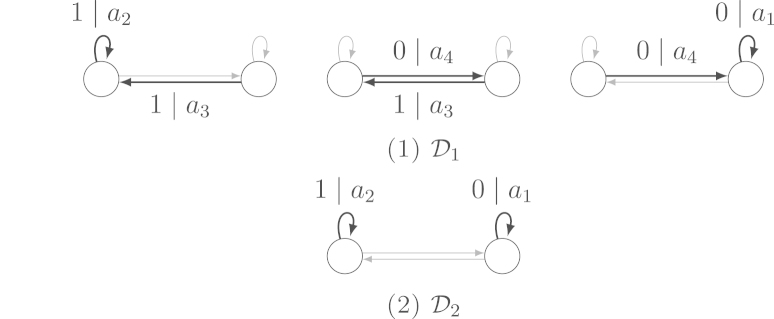
Functional digraphs of the transducer of Example 3.16.

Example 3.16 We again consider the transducer of Example 3.11 in Fig. 3. The set $D_{1}$ consists of 3 functional digraphs and $D_{2}$ consists of only one functional digraph (see Fig. 4). By  (7), we obtain the same equation as before, namely

$$a_{1}-a_{2}=0\text{,}$$

as condition for the transducer to be independent.

Also by Theorem 3.14, the expected value of the output sum is

$$\frac{a_{1}+a_{2}+a_{3}+a_{4}}{4}n+O\left(1\right)$$

and the asymptotic variance is

$$\frac{5a_{1}^{2}-6a_{1}a_{2}+5a_{2}^{2}-2a_{1}a_{3}-2a_{2}a_{3}+a_{3}^{2}-2a_{1}a_{4}-2a_{2}a_{4}+2a_{3}a_{4}+a_{4}^{2}}{16}\text{.}$$

The covariance between the input sum and the output sum is

$$-\frac{a_{1}-a_{2}}{4}n+O\left(1\right)\text{.}$$

## 4 Examples of transducers

In this section we give various examples to illustrate our theorems: these include both dependent and independent transducers and transducers with both bounded and unbounded variance of the output sum. These examples are also shown in the documentation of the method FiniteStateMachine.asymptotic_moments()   [17] in SageMath. Example 4.6 demonstrates how the combinatorial characterization of transducers with bounded variance can be used in cases where we only have limited information about the transducer.

Example 4.1 **Width-w Non-Adjacent Form**

**Fig. 5:**
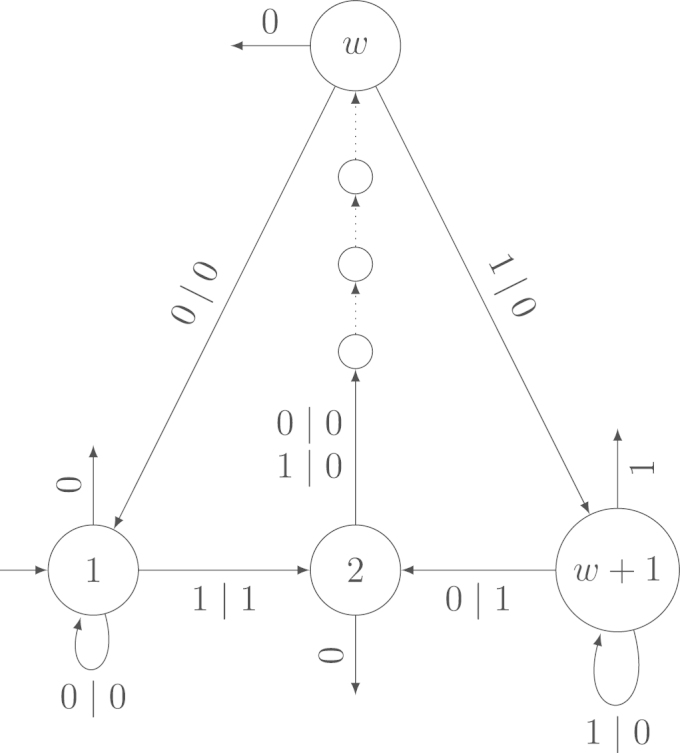
Transducer to compute the Hamming weight of the width-w non-adjacent form.

The width-w non-adjacent form (cf.  [1], [22]) is a digit expansion with base 2, digits $\left\{0,\pm 1,\pm 3,\ldots ,\pm \left(2^{w-1}-1\right)\right\}$ and the syntactical rule that at most one of any w consecutive digits is nonzero. The transducer in Fig. 5 computes the Hamming weight of the width-w non-adjacent form when reading the standard binary expansion (cf.  [16]). For w=2, this transducer is the same as that in Fig. 2. The variance of the output is not 0 (Corollary 3.6). With Theorem 3.9 or  3.14, we obtain that this transducer is independent for every w. Thus, the Hamming weight of the width-w non-adjacent form and the standard binary expansion are asymptotically independent.

Remark 4.2 Example 4.1 not only shows that there are infinitely many independent transducers, but also gives the construction of one such infinite family of independent transducers.

Example 4.3 **Gray Code**

**Fig. 6:**
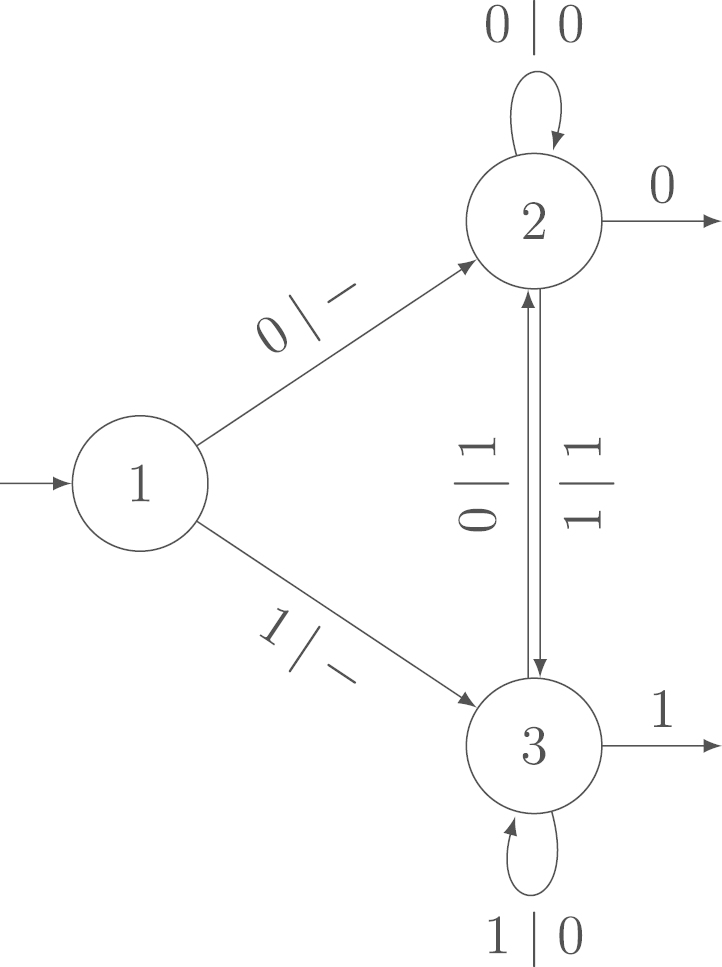
Transducer to compute the Gray code.

The Gray code is an encoding of the positive integers such that the Gray code of n and the Gray code of n+1 differ only at one position. The transducer in Fig. 6 computes the Gray code of an integer. The output label of the initial state is 0 and, as it does not influence the result, it is not given in the figure. The transducer is finally connected and finally aperiodic. The final component consisting of states 2 and 3 is independent (see Example 3.11). Thus, the Hamming weight of the Gray code and the standard binary expansion are asymptotically independent.

Example 4.4 **Length 2 Blocks in the Standard Binary Expansion**

**Fig. 7:**
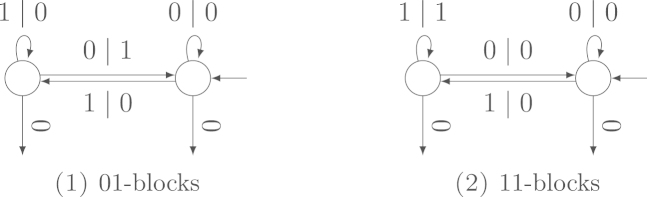
Transducers to count the number of 01- and 11-blocks in the standard binary expansion.

We count the number of patterns of length 2 occurring in the standard binary expansion and compare it to the Hamming weight. By symmetry, it is obviously sufficient to consider the two patterns 01 and 11. The transducers in Fig. 7 determine the number of 01- and 11-blocks, respectively. The variance of the output weight is not 0 in either case (Corollary 3.6), in fact the constant $v_{2}$ is $\frac{1}{16}$ (for 01-blocks) and $\frac{5}{16}$ respectively.

By Theorem 3.9 or  3.14, we also find that the transducer for 01-blocks is independent, while the transducer for 11-blocks (unsurprisingly) is not: the number of 11-blocks asymptotically depends on the number of 1’s in the standard binary expansion, and the correlation coefficient is $\frac{2}{\sqrt{5}}\approx 0.894$.

**Fig. 8:**
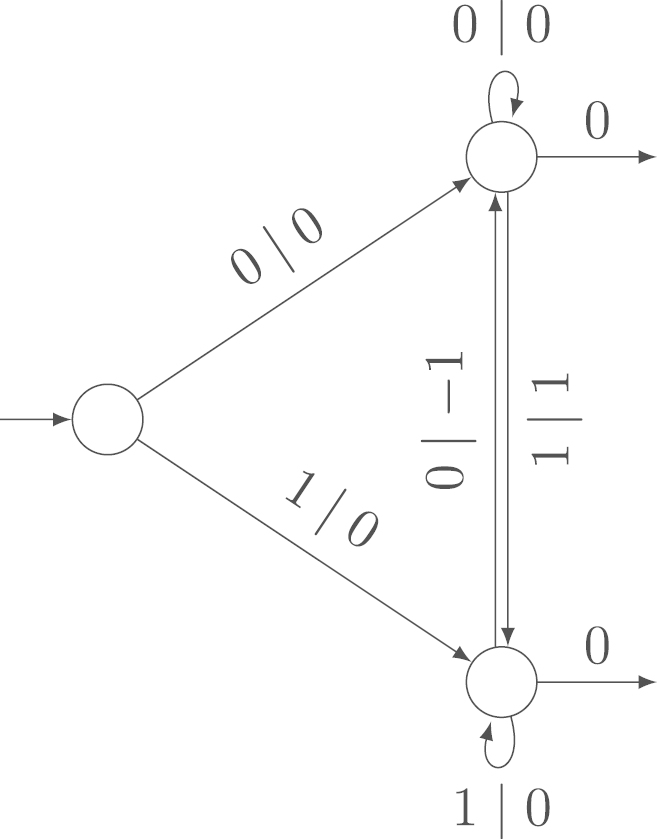
Transducer to compute the number of 10-blocks minus the number of 01-blocks in the standard binary expansion.

Example 4.5 Now, we give an example of a transducer with bounded variance of the output sum. We compute the number of 10-blocks minus the number of 01-blocks in the standard binary digit expansion. In Fig. 8, we show the corresponding transducer. The output label of the initial state is 0 and, as it does not influence the result, it is not given in the figure. Any of the three cycles has output sum 0. Thus, the asymptotic variance of this random variable is 0. There is, of course, an intuitive explanation: when we read a 1 after a 0 (reading from right to left), the count increases by 1; when we read a 0 after a 1, the count decreases by 1; otherwise, it remains unchanged. Thus the final output value will only depend on the first and last digit.

Example 4.6 Finally, we consider the minimal Hamming weight of τ-adic digit expansions for a given algebraic integer τ and a given digit set D. For z∈Z[τ], a τ-adic expansion ${\left(d_{L}\ldots d_{0}\right)}_{\tau }$ of z with digit set D⊂Z[τ] satisfies $d_{i}\in D$ and

$$z=\overset{L}{\underset{i=0}{\sum }}d_{i}\tau^{i}\text{.}$$

This can be extended to d-dimensional joint expansions of vectors $z\in Z{\left[\tau \right]}^{d}$ with digit set $D\subset Z{\left[\tau \right]}^{d}$.

In  [13], a transducer to compute the minimal Hamming weight is constructed. Note that the output alphabet of the transducer need not be {0,1} even if we are interested in the Hamming weight. The next theorem is an extension of Theorem 4 in  [13].

Theorem 4.7 *Assume that* $D\subset Z{\left[\tau \right]}^{d}$*, for a positive integer* d*, and* $D\cap \tau Z^{d}=\left\{0\right\}$ *. Let* mw(z) *be the minimal Hamming weight of a* τ*-adic joint digit representation of* z *with digits in* D *. Assume further that the digit set* D *satisfies*

$$\forall c\in Z{\left[\tau \right]}^{d}\;\exists U\in R\;\forall z\in Z{\left[\tau \right]}^{d}:\left|\mathrm{mw}\left(z+c\right)-\mathrm{mw}\left(z\right)\right|\leq U\text{.}$$

*Consider the random variable* $R_{n}=\mathrm{mw}\left(D_{n}\right)$*, where* $D_{n}$ *is a random* τ*-adic joint digit representation of length* n *with digits in* $A_{I}\subset Z{\left[\tau \right]}^{d}$ *. We assume that* $\left(\tau ,A_{I}\right)$ *is an irredundant digit system with* $0\in A_{I}$ *. The digits of* $D_{n}$ *are independent and identically distributed with uniform distribution on* $A_{I}$*.*

*Then there exist constants* E*,* V*, with* V≠0*, such that*

$$ER_{n}=En+O\left(1\right)\text{,}$$

$$VR_{n}=Vn+O\left(1\right)$$

*and*

$$\frac{R_{n}-En}{\sqrt{Vn}}$$

*is asymptotically normally distributed.*

Proof In  [13], the authors give a strongly connected and aperiodic transducer computing mw(z) if the input is the τ-adic representation of z with digit set $A_{I}$ read from left to right. Everything follows from Theorem 4 in  [13] if V≠0.

To prove V≠0, we use Theorem 3.1, (b). In  [13], the authors state that the transducer has a loop at the initial state 1 with input and output digit 0. Thus, in Theorem 3.1, (b), the value of k is 0.

On the other hand, there exists a $z\in Z{\left[\tau \right]}^{d}$ with mw(z)≠0. The input z leads to a state s. From each state the input $0^{l}$, for some l, leads again to the initial state 1. Thus, the unique path whose input labels are given by the digit representation of $z\tau^{l}$ is a closed walk visiting 1 at least once. The output sum of this closed walk is $\mathrm{mw}\left(z\tau^{l}\right)=\mathrm{mw}\left(z\right)\neq 0$. Thus, there exists a closed walk whose output sum is not 0, which contradicts Theorem 3.1, (b) with k=0. Therefore, we obtain V≠0. □

## 5 Proofs of the theorems

In this section, we give the proofs of the theorems and corollaries of Section  3. We first prove the algebraic description and the combinatorial characterization in Sections  3.2, 3.3. Later we prove the statements in Section  3.1 about the bounded variance.

### 5.1 Algebraic description of independent transducers

First, we prove a slight extension of the 2-dimensional Quasi-Power Theorem  [14] (a generalization of  [20]). This extension will also take into account the case of a singular Hessian matrix.

We write boldface letters for a vector $s={\left(s_{1},s_{2}\right)}^{t}$. Furthermore, we use the notation $e^{s}=\left(e^{s_{1}},e^{s_{2}}\right)$. We denote by 1 a 2- or 3-dimensional vector of ones, depending on the context. By ‖⋅‖, we denote the maximum norm $\left\|s\right\|=\max\left(\left|s_{1}\right|,\left|s_{2}\right|\right)$.

Theorem 5.1 *Let* ${\left(\Omega_{n}\right)}_{n\geq 1}$ *be a sequence of*  2*-dimensional real random vectors. Suppose that the moment generating function satisfies*

$$E\left(e^{\langle \Omega_{n},s\rangle }\right)=e^{u\left(s\right)\Phi \left(n\right)+v\left(s\right)}\left(1+O\left(\kappa_{n}^{-1}\right)\right)\text{,}$$

*the* O*-term being uniform for* ‖s‖≤τ*,* $s\in C^{2}$*,* τ>0*, where*

(1) u(s) *and* v(s) *are analytic for* ‖s‖≤τ *and independent of* n *;*
(2) $\lim_{n\to \infty }\Phi \left(n\right)=\infty$ *;*
(3) $\lim_{n\to \infty }\kappa_{n}=\infty$*.*

*Then,*

(8) $$E\left(\Omega_{n}\right)=\Phi \left(n\right)\mathrm{grad}\;u\left(0\right)+\mathrm{grad}\;v\left(0\right)+O\left(\kappa_{n}^{-1}\right)\text{,}$$

$$V\left(\Omega_{n}\right)=\Phi \left(n\right)H_{u}\left(0\right)+H_{v}\left(0\right)+O\left(\kappa_{n}^{-1}\right)\text{,}$$

*where* $H_{u}\left(s\right)$ *is the Hessian matrix of* u *. Let* Σ *be the matrix* $H_{u}\left(0\right)$*.*

*If* $H_{u}\left(0\right)$ *is regular, then the standardized random vector*

$$\Omega_{n}^{*}=\frac{\Omega_{n}-\Phi \left(n\right)\mathrm{grad}\;u\left(0\right)}{\sqrt{\Phi \left(n\right)}}$$

*is asymptotically jointly normally distributed with variance–covariance matrix* Σ*.*

*If* $H_{u}\left(0\right)$ *has rank*  1*, then the limit distribution of* $\Omega_{n}^{*}$ *is the direct product of a normal distribution and a degenerate distribution (if one of the variances is* O(1) *) or a linear transformation thereof. In the first case, the coordinates of* $\Omega_{n}^{*}$ *are asymptotically independent. In the second case, we have an asymptotically linear relationship between the two coordinates.*

*If* $H_{u}\left(0\right)$ *has rank*  0*, then the limit distribution of* $\Omega_{n}^{*}$ *is degenerate.*

Proof The expressions (8) for expectation and variance–covariance matrix follow from the moment generating function by differentiation.

The case of a regular Hessian matrix $H_{u}\left(0\right)$ is exactly the statement of the 2-dimensional Quasi-Power Theorem  [14].

For the case of a singular Hessian matrix, we follow the proof of the Quasi-Power Theorem  [14]. We consider the characteristic function

$$f_{n}\left(s\right)=\exp\left(-\frac{1}{2}s^{t}H_{u}\left(0\right)s+O\left(\frac{{\left\|s\right\|}^{3}+\left\|s\right\|}{\sqrt{\Phi \left(n\right)}}\right)\right)\left(1+O\left(\kappa_{n}^{-1}\right)\right)$$

of the standardized random vector $\Omega_{n}^{*}$. Thus the characteristic function tends to

$$f\left(s\right)=\exp\left(-\frac{1}{2}s^{t}H_{u}\left(0\right)s\right)\text{.}$$

If the Hessian matrix $H_{u}\left(0\right)$ has rank 0, then f(s) equals the identity function. Thus, the distribution function is degenerate.

If the Hessian matrix $H_{u}\left(0\right)$ has rank 1 and the variance of the second coordinate $\Omega_{n,2}$ is O(1), then $H_{u}\left(0\right)=\left(\begin{array}{cc} v_{1} & 0\\ 0 & 0 \end{array}\right)$ for a $v_{1}\in R$. Thus,

$$f\left(s\right)=\exp\left(-\frac{1}{2}v_{1}^{2}s_{1}^{2}\right)\cdot 1$$

which is the characteristic function of the normal distribution with mean 0 and variance $v_{1}$ times the characteristic function of the point mass at 0.

If the Hessian matrix $H_{u}\left(0\right)=\left(\begin{array}{cc} v_{1} & c\\ c & v_{2} \end{array}\right)$ has rank 1 with $v_{1}v_{2}\neq 0$, then we consider the random variables $X=\Omega_{n,1}$, the first coordinate of $\Omega_{n}$, and $Z=-\frac{c}{v_{1}}\Omega_{n,1}+\Omega_{n,2}$. Then, the main term of the variance–covariance matrix of ${\left(X,Z\right)}^{t}$ is $\left(\begin{array}{cc} v_{1} & 0\\ 0 & 0 \end{array}\right)\Phi \left(n\right)$. Thus, X is asymptotically normally distributed and Z is an asymptotically constant random variable (see previous case). □

Using this version of the Quasi-Power Theorem, we prove the algebraic description of independent transducers given in Theorem 3.9.

Proof of Theorem 3.9 Let K be the size of the input alphabet $A_{I}$. Let $a_{kln}$ be the number of sequences of length n with input sum k such that the corresponding output of the transducer T has sum l. We define

$$A\left(x,y,z\right)=\underset{k\in R}{\sum }\underset{l\in R}{\sum }\overset{\infty }{\underset{n=0}{\sum }}a_{kln}K^{-n}x^{k}y^{l}z^{n}\text{.}$$

Thus, the variable x marks the input sum, y marks the output sum, and z marks the length of the input. Then $\left[z^{n}\right]A\left(x,y,z\right)$ is the probability generating function of $\Omega_{n}$, where $\left[z^{n}\right]b\left(z\right)$ is the coefficient of $z^{n}$ in the power series b(z).

Due to the block structure of $W_{\varepsilon }\left(y\right)$ in (2), we have

(9) $$A\left(x,y,z\right)=u^{t}{\left(I-\frac{z}{K}\underset{\varepsilon \in A_{I}}{\sum }x^{\varepsilon }W_{\varepsilon }\left(y\right)\right)}^{-1}v=\frac{F_{1}\left(x,y,z\right)}{\det\left(I-\frac{z}{K}\underset{\varepsilon \in A_{I}}{\sum }x^{\varepsilon }W_{\varepsilon }\left(y\right)\right)}=\frac{F_{1}\left(x,y,z\right)}{F_{2}\left(x,y,z\right)\det\left(I-\frac{z}{K}\underset{\varepsilon \in A_{I}}{\sum }x^{\varepsilon }M_{\varepsilon }\left(y\right)\right)}\text{,}$$

with $u^{t}=\left(1,0,\ldots ,0\right)$ for the initial state, $v_{s}=y^{a\left(s\right)}$ for the final output label at state s and $F_{1}\left(x,y,z\right)$ and $F_{2}\left(x,y,z\right)$ “polynomials” in x, y and z. We use quotation marks because exponents of x and y might not be integers. However, only finitely many summands occur. The function $F_{2}\left(x,y,z\right)$ is the determinant corresponding to the non-final components in the upper left corner in (2).

The moment generating function of $\Omega_{n}$ is

$$E\left(e^{\langle \Omega_{n},s\rangle }\right)=\left[z^{n}\right]A\left(e^{s_{1}},e^{s_{2}},z\right)\text{.}$$

For extracting the coefficient, we investigate the dominant singularity of A(x,y,z). Since the final component is strongly connected and aperiodic, we have a unique dominant simple eigenvalue of $\sum_{\varepsilon \in A_{I}}x^{\varepsilon }M_{\varepsilon }\left(y\right)$ at ${\left(x,y\right)}^{t}=1$ by the theorem of Perron–Frobenius (cf.  [7]). Because the final component is complete, this dominant eigenvalue is K, that is the size of the input alphabet $A_{I}$. Thus, the unique dominant singularity of $f{\left(x,y,z\right)}^{-1}=\det{\left(I-\frac{z}{\left|A_{I}\right|}\sum_{\varepsilon \in A_{I}}x^{\varepsilon }M_{\varepsilon }\left(y\right)\right)}^{-1}$ at ${\left(x,y\right)}^{t}=1$ is a simple pole at ρ(1)=1. Therefore, we have $f_{z}\left(1\right)\neq 0$.

For ${\left(x,y\right)}^{t}$ in a small neighborhood of 1, there is a unique dominant singularity ρ(x,y) of $f{\left(x,y,z\right)}^{-1}$ due to the continuity of eigenvalues.

Next, we consider the non-final components of the transducer. The corresponding transducer $T_{0}$ is not complete. Let $T_{0}^{+}$ be the complete transducer that is obtained from $T_{0}$ by adding loops where necessary. The dominant eigenvalue of $T_{0}^{+}$ is K. As the corresponding sums of transition matrices of $T_{0}$ and $T_{0}^{+}$ satisfy element-wise inequalities but are not equal (at ${\left(x,y\right)}^{t}=1$), the theorem of Perron–Frobenius (cf.  [7, Theorem 8.8.1]) implies that the dominant eigenvalues of $T_{0}$ have absolute value less than K. Thus, the dominant singularities of $F_{2}{\left(1,1,z\right)}^{-1}$ are at |z|>1. By continuity, this also holds for a small neighborhood of ${\left(x,y\right)}^{t}=1$.

As $A\left(1,1,z\right)={\left(1-z\right)}^{-1}$, we obtain $F_{1}\left(1\right)\neq 0$ and $F_{1}\left(x,y,\rho \left(x,y\right)\right)\neq 0$ for ${\left(x,y\right)}^{t}$ in a small neighborhood of 1. Therefore, ρ(x,y) is the simple dominant pole of A(x,y,z) in a small neighborhood of 1.

The Laurent series of A(x,y,z) at z=ρ(x,y) is

$$A\left(x,y,z\right)={\left(z-\rho \left(x,y\right)\right)}^{-1}C\left(x,y\right)+\text{power series in }\left(z-\rho \left(x,y\right)\right)$$

for a function C(x,y) which is analytic in a neighborhood of 1 with C(1)≠0. Thus, by singularity analysis  [5], we have

$$E\left(e^{\langle \Omega_{n},s\rangle }\right)=\left[z^{n}\right]A\left(e^{s_{1}},e^{s_{2}},z\right)=e^{u\left(s\right)n+v\left(s\right)}\left(1+O\left(\kappa^{n}\right)\right)$$

with

$$u\left(s\right)=-\log\rho \left(e^{s}\right)\text{,}$$

$$v\left(s\right)=\log\left(-C\left(e^{s}\right)\rho {\left(e^{s}\right)}^{-1}\right)$$

and κ<1.

Theorem 5.1 yields the expected value, the variance–covariance matrix and the asymptotic normality of $\Omega_{n}$. By implicit differentiation, we obtain the stated expressions. The error terms for the input sum are 0 because the input letters are independent and identically distributed. This also yields the explicit constants in (4).

Since the input alphabet $A_{I}$ has at least two elements, the input sum has nonzero asymptotic variance. Thus, the asymptotic variance–covariance matrix Σ can have rank 1 or 2. Now, we consider these two cases separately and prove the asserted equivalence.

(1) Let Σ have rank 1. Then $\Omega_{n}$ converges to a degenerate and a normally distributed random variable if the asymptotic variance of the output sum is 0; or a linear transformation thereof otherwise. Thus, $\Omega_{n}$ is asymptotically independent if and only if the asymptotic variance of the sum of the output is 0. As the rank of Σ is 1, the asymptotic variance is 0 if and only if the asymptotic covariance is 0.
(2) Let Σ be invertible. By Theorem 5.1, we obtain an asymptotic joint normal distribution. Thus, $\Omega_{n}$ is asymptotically independent if and only if its asymptotic covariance is 0. □

### 5.2 Combinatorial characterization of independent transducers

To obtain the combinatorial characterization, we use a version of the Matrix-Tree Theorem as proved by Chaiken  [4] and Moon  [21]. This version does not use trees, but *forests*, i.e., digraphs whose weak components are trees.

Definition 5.2 Let A, B⊆{1,…,N}. Let $F_{A,B}$ be the set of all forests which are spanning subgraphs of the final component of the transducer T with |A| trees such that every tree is rooted at some vertex a∈A and contains exactly one vertex b∈B.

Let $A=\left\{i_{1},\ldots ,i_{n}\right\}$ and $B=\left\{j_{1},\ldots ,j_{n}\right\}$ with $i_{1}<\cdots <i_{n}$ and $j_{1}<\cdots <j_{n}$. For $F\in F_{A,B}$, we define a function g:B→A by g(j)=i if j is in the tree of F which is rooted in vertex i. We further define the function h:A→B by $h\left(i_{k}\right)=j_{k}$ for k=1,…,n. The composition g∘h:A→A is a permutation on A. We define signF=signg∘h.

If |A|≠|B|, then $F_{A,B}=0̸$. If |A|=|B|=1, then signF=1 and $F_{A,B}$ consists of all spanning trees rooted in a∈A.

Theorem **All-Minors-Matrix-Tree Theorem [4], [21]**

*For a directed graph with loops, let* $L={\left(l_{ij}\right)}_{1\leq i,j\leq N}$ *be the Laplacian matrix, that is* $\sum_{j=1}^{N}l_{ij}=0$ *for every* i=1,…,N *and* $-l_{ij}$ *is the number of edges from* i *to* j *for* i≠j *. Then, for* |A|=|B|*, the minor* $\det L_{A,B}$ *satisfies*

$$\det L_{A,B}={\left(-1\right)}^{\underset{i\in A}{\sum }i+\underset{j\in B}{\sum }j}\underset{F\in F_{A,B}}{\sum }\mathrm{sign}\;F$$

*where* $L_{A,B}$ *is the matrix* L *whose rows with index in* A *and columns with index in* B *are deleted.*

The All-Minors-Matrix-Tree Theorem is still valid for |A|≠|B| if we assume that the determinant of a non-square matrix is 0. For notational simplicity, we use this convention in the rest of this section.

The next lemma connects the derivatives of f(x,y,z) with weighted sums of functional digraphs. Theorem 3.14 follows immediately from this lemma and Theorem 3.9.

Lemma 5.3 *Let* K *be the size of the input alphabet* $A_{I}$ *. For* $f\left(x,y,z\right)=\det\left(I-\frac{z}{\left|A_{I}\right|}\sum_{\varepsilon \in A_{I}}x^{\varepsilon }M_{\varepsilon }\left(y\right)\right)$*, we have*

$$f_{x}\left(1,1,1\right)=-K^{-N}\varepsilon \left(D_{1}\right)\text{,}\;f_{xy}\left(1,1,1\right)=K^{-N}\left(\varepsilon \delta \left(D_{2}\right)-\varepsilon \delta \left(D_{1}\right)\right)\text{,}$$

$$f_{y}\left(1,1,1\right)=-K^{-N}\delta \left(D_{1}\right)\text{,}\;f_{xz}\left(1,1,1\right)=K^{-N}\left(\varepsilon 1\left(D_{2}\right)-\varepsilon 1\left(D_{1}\right)\right)\text{,}$$

$$f_{z}\left(1,1,1\right)=-K^{-N}1\left(D_{1}\right)\text{,}\;f_{yz}\left(1,1,1\right)=K^{-N}\left(\delta 1\left(D_{2}\right)-\delta 1\left(D_{1}\right)\right)\text{,}$$

$$f_{xx}\left(1,1,1\right)+f_{x}\left(1,1,1\right)=K^{-N}\left(\varepsilon \varepsilon \left(D_{2}\right)-\varepsilon \varepsilon \left(D_{1}\right)\right)\text{,}$$

$$f_{yy}\left(1,1,1\right)+f_{y}\left(1,1,1\right)=K^{-N}\left(\delta \delta \left(D_{2}\right)-\delta \delta \left(D_{1}\right)\right)\text{,}$$

$$f_{zz}\left(1,1,1\right)+f_{z}\left(1,1,1\right)=K^{-N}\left(11\left(D_{2}\right)-11\left(D_{1}\right)\right)\text{.}$$

Proof The idea of the proof is as follows: First, we compute the derivatives and write them as sums over all states. Using the All-Minors-Matrix-Tree Theorem, we change the summation to a sum over forests. In the next step, we again change to a sum over functional digraphs.

Let $u_{1}$, $u_{2}$ be any of the variables x, y or z. For a matrix $M={\left(m_{ij}\right)}_{1\leq i,j\leq N}$, we define the matrix $M_{k:u_{1}}={\left({\overset{ˆ}{m}}_{ij}\right)}_{1\leq i,j\leq N}$ with ${\overset{ˆ}{m}}_{ij}=m_{ij}$ for i≠k and ${\overset{ˆ}{m}}_{kj}=\frac{\partial }{\partial u_{1}}m_{kj}$. Thus $M_{k:u_{1}}$ is the matrix M where row k is differentiated with respect to $u_{1}$.

We further define the derivatives at 1 as

$$D_{u_{1}}\left(\;\cdot \;\right)=\frac{\partial }{\partial u_{1}}\left(\;\cdot \;\right){|}_{1}$$

and

$$D_{u_{1}u_{2}}\left(\;\cdot \;\right)=\frac{\partial^{2}}{\partial u_{1}\partial u_{2}}\left(\;\cdot \;\right){|}_{1}\text{.}$$

Applying the product rule to the definition of the determinants gives us

$$D_{u_{1}}\left(f\right)=\overset{N}{\underset{j=1}{\sum }}\det{\left(I-\frac{z}{K}\underset{\varepsilon \in A_{I}}{\sum }x^{\varepsilon }M_{\varepsilon }\left(y\right)\right)}_{j:u_{1}}{|}_{1}\text{,}$$

$$D_{u_{1}u_{2}}\left(f\right)=\overset{N}{\underset{i=1}{\sum }}\overset{N}{\underset{j=1}{\sum }}\det{\left(I-\frac{z}{K}\underset{\varepsilon \in A_{I}}{\sum }x^{\varepsilon }M_{\varepsilon }\left(y\right)\right)}_{i:u_{1},\;j:u_{2}}{|}_{1}\text{.}$$

In these equations, we have a sum over all states.

Since our original matrix $I-\frac{z}{K}\sum_{\varepsilon \in A_{I}}x^{\varepsilon }M_{\varepsilon }\left(y\right)$ is sparse, and ${\left(I-\frac{z}{K}\sum_{\varepsilon \in A_{I}}x^{\varepsilon }M_{\varepsilon }\left(y\right)\right)}_{j:u_{1}}$ is even sparser, we use Laplace expansion along row j to determine these determinants. If i≠j, we use Laplace expansion along row i and j to determine $\det{\left(I-\frac{z}{K}\sum_{\varepsilon \in A_{I}}x^{\varepsilon }M_{\varepsilon }\left(y\right)\right)}_{i:u_{1},\;j:u_{2}}$ for the second derivatives. If i=j, we only expand along row j. Depending on the variable of differentiation, there are at most K nonzero values in row j after differentiation.

For a transition e, we denote by t(e), h(e), ε(e) and δ(e) the tail, the head, the input and the output of the transition e, respectively. Furthermore, let $w_{e}=\frac{1}{K}x^{\varepsilon \left(e\right)}y^{\delta \left(e\right)}z$ be the weight of the transition e.

If we use Laplace expansion along two different rows, we must be careful with the sign. Therefore, we define

$$\sigma_{de}={\left(-1\right)}^{\left[t\left(e\right)>t\left(d\right)\right]+\left[h\left(e\right)>h\left(d\right)\right]}$$

for two transitions d and e. Here, we use Iverson’s notation, that is [*expression*] is 1 if *expression* is true and 0 otherwise (cf.  [12]).

Let L be the Laplacian matrix of the underlying graph, that is

$$L=KI-\underset{\varepsilon \in A_{I}}{\sum }M_{\varepsilon }\left(1\right)\text{.}$$

Recall the notation $L_{A,B}$ for the matrix where the rows corresponding to A and the columns corresponding to B have been removed. Laplace expansion yields

Du1(f)=−K−N+1∑j=1N∑e∈Et(e)=j(−1)t(e)+h(e)Du1(we)det(L{t(e)},{h(e)}),

Du1u2(f)=−K−N+1∑j=1N∑e∈Et(e)=j(−1)t(e)+h(e)Du1u2(we)det(L{t(e)},{h(e)})+K−N+2∑i=1N∑j=1j≠iN∑d∈Et(d)=i∑e∈Et(e)=j((−1)t(d)+h(d)+t(e)+h(e)σdeDu1(wd)Du2(we)det(L{t(d),t(e)},{h(d),h(e)})).

Next, we use the All-Minors-Matrix-Tree Theorem and change the summation over all rows to a summation over forests. We obtain

$$D_{u_{1}}\left(f\right)=-K^{-N+1}\underset{e\in E}{\sum }D_{u_{1}}\left(w_{e}\right)\underset{F\in F_{\left\{t\left(e\right)\right\},\left\{h\left(e\right)\right\}}}{\sum }\mathrm{sign}\;F\text{,}$$

Du1u2(f)=−K−N+1∑e∈EDu1u2(we)∑F∈F{t(e)},{h(e)}signF+K−N+2∑d∈E∑e∈Ee≠d(σdeDu1(wd)Du2(we)∑F∈F{t(d),t(e)},{h(d),h(e)}signF).

Let $F\in F_{\left\{t\left(e\right)\right\},\left\{h\left(e\right)\right\}}$ be a forest for a transition e∈E. Then F+e is a spanning functional digraph with one component. Let $F\in F_{\left\{t\left(d\right),t\left(e\right)\right\},\left\{h\left(d\right),h\left(e\right)\right\}}$ be a forest for transitions d,e∈E. Then F+d+e is a spanning functional digraph with one or two components, depending on $\sigma_{de}\mathrm{sign}\;F$. If $\sigma_{de}\mathrm{sign}\;F=1$, then it has two components. Otherwise, it has one component. Now we can change the summation into a sum over functional digraphs and obtain

$$D_{u_{1}}\left(f\right)=-K^{-N+1}\underset{D\in D_{1}}{\sum }\underset{C\in C_{D}}{\sum }\underset{e\in C}{\sum }D_{u_{1}}\left(w_{e}\right)\text{,}$$

Du1u2(f)=−K−N+1∑D∈D1∑C∈CD∑e∈CDu1u2(we)+K−N+2∑D∈D2∑C1∈CD∑C2∈CDC2≠C1∑d∈C1∑e∈C2Du1(wd)Du2(we)−K−N+2∑D∈D1∑C∈CD∑d∈C∑e∈Ce≠dDu1(wd)Du2(we).

For a transition e, we know the first derivatives

$$D_{x}\left(w_{e}\right)=\frac{1}{K}\varepsilon \left(e\right)\text{,}\;D_{y}\left(w_{e}\right)=\frac{1}{K}\delta \left(e\right)\text{,}\;D_{z}\left(w_{e}\right)=\frac{1}{K}1\left(e\right)\text{,}$$

and the second derivatives

$$D_{xy}\left(w_{e}\right)=\frac{1}{K}\varepsilon \left(e\right)\delta \left(e\right)\text{,}\;D_{xx}\left(w_{e}\right)=\frac{1}{K}\varepsilon \left(e\right)\left(\varepsilon \left(e\right)-1\right)\text{,}$$

$$D_{xz}\left(w_{e}\right)=\frac{1}{K}\varepsilon \left(e\right)1\left(e\right)\text{,}\;D_{yy}\left(w_{e}\right)=\frac{1}{K}\delta \left(e\right)\left(\delta \left(e\right)-1\right)\text{,}$$

$$D_{yz}\left(w_{e}\right)=\frac{1}{K}\delta \left(e\right)1\left(e\right)\text{,}\;D_{zz}\left(w_{e}\right)=0\text{.}$$

Thus, we obtain the formulas stated in the lemma. □

### 5.3 Bounded variance and singular asymptotic variance–covariance matrix

We next give the proof of the equivalence of the three statements in Theorem 3.1, including the bounded variance.

Proof of Theorem 3.1 We first prove (a) ⇔ (b) by giving an alternative representation of the generating function A(x,y,z) from the proof of Theorem 3.9. Then we prove the equivalence (b) ⇔ (c).

(a) ⇔(b) WLOG, we assume that the expected value $E\left(\mathrm{Output}\left(X_{n}\right)\right)$ is a O(1). Otherwise, we have $E\left(\mathrm{Output}\left(X_{n}\right)\right)=e_{2}n+O\left(1\right)$ for some constant $e_{2}$ (see Theorem 3.9). Then we subtract $e_{2}$ from the output of every transition, as for Corollary 3.15. Under this assumption, Theorem 3.14 implies that (b) can only hold with k=0.
As the input sum is inconsequential, we consider A(1,y,z). For brevity, we write A(y,z) instead. We obtain
  $$A\left(y,z\right)=u^{t}{\left(I-\frac{z}{K}\underset{\varepsilon \in A_{I}}{\sum }W_{\varepsilon }\left(y\right)\right)}^{-1}v$$
  where $W_{\varepsilon }$ for ε∈{0,…,q−1} are the transition matrices of T and $K=\left|A_{I}\right|$.
Since T is complete, finally connected and finally aperiodic, A(1,z) has a simple dominant pole at z=1 (see the proof of Theorem 3.9). We know that
  (10) $$E\left(\mathrm{Output}\left(X_{n}\right)\right)=\left[z^{n}\right]A_{y}\left(1,z\right)=O\left(1\right)\text{,}$$
  $$V\left(\mathrm{Output}\left(X_{n}\right)\right)=\left[z^{n}\right]A_{yy}\left(1,z\right)+O\left(1\right)\text{.}$$
  Let s be any state of the final component. Each path starting at state 1 either does or does not visit state s. In the first case, this path can be decomposed into a path leading to state s and visiting s only once, followed by a sequence of closed walks visiting state s exactly once, and a path starting in s and not returning to s. We translate this decomposition into an equation for the corresponding generating functions.
  Let $P^{s}$ be the set of all walks in T which start at state s but never return to state s. All other states can be visited arbitrarily often. We define the corresponding generating function $P^{s}\left(y,z\right)=\sum_{P\in P^{s}}y^{\delta \left(P\right)}z^{1\left(P\right)}K^{-1\left(P\right)}$. Then $\left[z^{n}\right]P^{s}\left(y,z\right)$ is the probability generating function of the output sum over walks in $P^{s}$ of length n.
  Let $P^{1s}$ be the set of all walks in T which start at state 1 and lead to state s, visiting s exactly once. If s=1, this set consists only of the path of length 0. The corresponding generating function is called $P^{1s}\left(y,z\right)$.
  Let $P^{1}$ be the set of all walks in T which start at state 1 and never visit state s. If s=1, this set is empty. The corresponding generating function is called $P^{1}\left(y,z\right)$.
  Let $C^{s}$ be the set of all closed walks in T which visit state s exactly once. All other states can be visited arbitrarily often. The corresponding generating function is called $C^{s}\left(y,z\right)$.
Thus, we have
  (11) $$A\left(y,z\right)=P^{1}\left(y,z\right)+\frac{P^{1s}\left(y,z\right)P^{s}\left(y,z\right)}{1-C^{s}\left(y,z\right)}\text{.}$$
  Let α be any of the superscripts 1, 1s or s. By deleting the transitions leading to s, we have
  $$P^{\alpha }\left(y,z\right)={\left(u^{\alpha }\right)}^{t}{\left(I-\frac{z}{K}\underset{\varepsilon \in A_{I}}{\sum }W_{\varepsilon }\left(y\right)E\right)}^{-1}v^{\alpha }\text{,}$$
  where E=diag(1,…,1,0,1,…,1) and $u^{\alpha }$ and $v^{\alpha }$ are fixed vectors. The position of the zero on the diagonal of E corresponds to the state s. The vectors $u^{\alpha }$ and $v^{\alpha }$ depend on α and may include the output of the transitions leading to s, but E is independent of α. Since we have the element-wise inequalities
  $$0\leq \underset{\varepsilon \in A_{I}}{\sum }W_{\varepsilon }\left(1\right)E\leq \underset{\varepsilon \in A_{I}}{\sum }W_{\varepsilon }\left(1\right)$$
  and $\sum_{\varepsilon \in A_{I}}W_{\varepsilon }\left(1\right)E\neq \sum_{\varepsilon \in A_{I}}W_{\varepsilon }\left(1\right)$, we know that the spectral radii satisfy
  $$\rho \left(\underset{\varepsilon \in A_{I}}{\sum }W_{\varepsilon }\left(1\right)E\right)<\rho \left(\underset{\varepsilon \in A_{I}}{\sum }W_{\varepsilon }\left(1\right)\right)=K$$
  due to the theorem of Perron–Frobenius (cf.  [7, Theorem 8.8.1]). Here, it is important that s lies in the final component. Thus, the dominant singularities of $P^{\alpha }\left(1,z\right)$ are at |z|>1. Furthermore, we know that $P^{s}\left(1,1\right)>0$ and $P^{1s}\left(1,1\right)>0$ by the definition as generating functions.
  Because z=1 is a simple pole of A(1,z), no pole of $P^{1}\left(1,z\right)$ and $P^{1s}\left(1,z\right)P^{s}\left(1,z\right)$, and $P^{1s}\left(1,1\right)P^{s}\left(1,1\right)\neq 0$, it is a simple root of $1-C^{s}\left(1,z\right)$ by (11). Thus, we can write $1-C^{s}\left(1,z\right)=\left(z-1\right)g\left(z\right)$ for a suitable function g(z) with g(1)≠0.
  By  (10),  (11) and singularity analysis  [5], we obtain
  $$O\left(1\right)=E\left(\mathrm{Output}\left(X_{n}\right)\right)=P^{1s}\left(1,1\right)P^{s}\left(1,1\right)C_{y}^{s}\left(1,1\right)g{\left(1\right)}^{-2}n+O\left(1\right)\text{.}$$
  Therefore, $C_{y}^{s}\left(1,1\right)=0$.
Similarly, we have
  (12) $$V\left(\mathrm{Output}\left(X_{n}\right)\right)=P^{1s}\left(1,1\right)P^{s}\left(1,1\right)C_{yy}^{s}\left(1,1\right)g{\left(1\right)}^{-2}n+O\left(1\right)\text{,}$$
  taking into account that $C_{y}^{s}\left(1,1\right)=0$.
  By  (12), $V\left(\mathrm{Output}\left(X_{n}\right)\right)=O\left(1\right)$ is equivalent to $C_{yy}^{s}\left(1,1\right)=0$, and thus, $C_{yy}^{s}\left(1,1\right)+C_{y}^{s}\left(1,1\right)=0$ as $C_{y}^{s}\left(1,1\right)=0$. By the definition of $C^{s}\left(y,z\right)$, this is equivalent to
  $$\underset{C\in C^{s}}{\sum }\delta {\left(C\right)}^{2}K^{-1\left(C\right)}=0\text{,}$$
  and thus δ(C)=0 for all $C\in C^{s}$.
(b) ⇒(c) Let $C^{s}$ be the set of all closed walks in the final component of T which visit state s exactly once. If D is any cycle of the final component of the transducer, then one of the following occurs.
  • No visits of state s: Let i be a vertex of D. Because the final component is strongly connected, there exists a closed walk $C\in C^{s}$ with s, i∈C. Let $D^{\prime }$ be the combined closed walk of D and C. Then, $D^{\prime }\in C^{s}$, and so we have
    $$\delta \left(D\right)=\delta \left(D^{\prime }\right)-\delta \left(C\right)=k1\left(D^{\prime }\right)-k1\left(C\right)=k1\left(D\right)\text{.}$$
  • One visit of state s: Then we have $D\in C^{s}$ and δ(D)=k1(D).
(c) ⇒(b) As a closed walk visiting s exactly once can be decomposed into cycles, this is obvious. □

Next, we prove the equivalence for the quasi-deterministic output sum.

Proof of Theorem 3.3

(d) ⇒(e) Let C be an arbitrary cycle of the transducer and P be a path from the initial state 1 to any state of the cycle. Let $z_{n}$ be the input sequence along the combined walk consisting of P and n times C. Then, by quasi-determinism and the definition of the output, we have
  $$k\left(1\left(P\right)+n1\left(C\right)\right)+O\left(1\right)=\mathrm{Output}\left(z_{n}\right)=\delta \left(P\right)+n\delta \left(C\right)+O\left(1\right)\text{.}$$
  Thus, n(δ(C)−k1(C)) is bounded by a constant depending on P and C, but independent of n. Therefore, we know that δ(C)=k1(C).
(e) ⇒(d) WLOG, we assume k=0 (replace δ(e) by δ(e)−k for all transitions e). For every $z\in A_{I}^{*}$, we have $\left|\mathrm{Output}\left(z\right)\right|\leq \sum_{e\in E}\left|\delta \left(e\right)\right|+\max_{s\in \left\{1,\ldots ,S\right\}}\left|a\left(s\right)\right|$ because all cycles have output sum 0 so that every transition contributes at most once to Output(z). Therefore, we have a quasi-deterministic random variable $\mathrm{Output}\left(X_{n}\right)=O\left(1\right)$. □

Now, we consider transducers whose output alphabet is {0,1} and prove that there are only trivial cases with a bounded variance.

Proof of Corollary 3.6 We know that the output digits (0,…,0) and (1,…,1) have asymptotic variance 0.

Assume that the asymptotic variance is 0. Let k be the constant given in Theorem 3.1. Then, we know k∈[0,1]. By the aperiodicity of the final component, there exist cycles $C_{1},\ldots ,C_{n}$ of coprime length and therefore integers $b_{1},\ldots ,b_{n}$ with

$$1=b_{1}1\left(C_{1}\right)+\cdots +b_{n}1\left(C_{n}\right)\text{.}$$

Thus,

$$k=b_{1}\delta \left(C_{1}\right)+\cdots +b_{n}\delta \left(C_{n}\right)\in Z$$

and hence, k∈{0,1}. Therefore, (0,…,0) and (1,…,1) are the only output digits with asymptotic variance 0. □

This last proof shows the equivalence of the statements in Corollary 3.7, including a transducer with a singular asymptotic variance–covariance matrix.

Proof of Corollary 3.7 WLOG, we assume that both expected values $E\left(\mathrm{Output}\left(X_{n}\right)\right)$ and $E\left(\mathrm{Input}\left(X_{n}\right)\right)$ are O(1).

We know that the asymptotic variance $v_{1}$ of the input is non-zero because $A_{I}$ consists of at least two elements. As in the last paragraph of the proof of Theorem 5.1, we consider the random variables $Y_{n}=\mathrm{Input}\left(X_{n}\right)$ and $Z_{n}=-\frac{c}{v_{1}}\mathrm{Input}\left(X_{n}\right)+\mathrm{Output}\left(X_{n}\right)$ and their variance–covariance matrix

$$\left(\begin{array}{cc} v_{1} & 0\\ 0 & v_{2}-\frac{c^{2}}{v_{1}} \end{array}\right)\text{.}$$

The matrix Σ is singular if and only if the asymptotic variance of $Z_{n}$ is 0.

Thus, we consider a transducer with the same input as the original transducer T for which the output of a transition e is $-\frac{c}{v_{1}}\varepsilon \left(e\right)+\delta \left(e\right)$. By Theorem 3.1, the output sum of this new transducer has asymptotic variance 0 if and only if there exists an m∈R such that

$$-\frac{c}{v_{1}}\varepsilon \left(C\right)+\delta \left(C\right)=m1\left(C\right)$$

for every cycle C of the final component. Since the expected value of $Z_{n}$ is O(1), we have m=0.

The second statement follows from Theorem 3.1. □
